# A Text Messaging-Based Smoking Cessation Program for Adult Smokers: Randomized Controlled Trial

**DOI:** 10.2196/jmir.2231

**Published:** 2012-12-27

**Authors:** Michele Ybarra, A Tülay Bağcı Bosi, Josephine Korchmaros, Salih Emri

**Affiliations:** ^1^Center for Innovative Public Health ResearchSan Clemente, CAUnited States; ^2^Hacettepe UniversityDepartment of Public HealthAnkaraTurkey; ^3^School of MedicineDepartment of Chest DiseasesHacettepe UniversityAnkaraTurkey

**Keywords:** smoking cessation, Middle East, text messaging, pilot study

## Abstract

**Background:**

Despite promising data in Western countries, there is a dearth of research into the efficacy of text messaging-based smoking cessation programs in other settings, including the Middle East, where smoking prevalence rates are higher.

**Objective:**

This paper reports cessation rates observed in SMS Turkey, a text messaging-based smoking cessation program for adult smokers in Ankara, Turkey.

**Methods:**

This study was a small-scale, parallel-group randomized controlled trial (RCT) conducted in Ankara, Turkey. Participants were adult daily smokers who were seriously thinking about quitting in the next 15 days and living in Ankara, Turkey. The text messaging intervention, SMS Turkey, provided 6 weeks of daily messages aimed at giving participants skills to help them quit smoking. Messages were sent in an automated fashion, except 2 days and 7 days after the initial quit day. On days 2 and 7, the research assistant manually assigned participants to content “paths” based on whether they were still not smoking or had relapsed. The control arm received a brochure that provided similar information about smoking cessation. The main outcome measure was self-reported 3-month sustained abstinence, verified by carbon monoxide (CO) readings. Neither participants nor researchers were blinded to arm assignment.

**Results:**

The 151 participants were randomly assigned to 1 of 2 groups: 76 to the SMS Turkey intervention group and 75 to the brochure control group. Using intention to treat, all 151 participants were included in analyses. Three-month cessation trends were not significantly higher in the intervention group: 11% intervention vs 5% control had quit (χ^2^_1_=1.4, *P*=.24; R^2^=2.0, 95% CI 0.62-6.3). When the sample was stratified by sex, female intervention participants (14%, n=5) were significantly more likely to have quit at 3 months than female control participants (0%, n=0; χ^2^_1_=3.7, *P*=.05). Among light smokers (ie, those smoking less than 20 cigarettes per day), intervention participants (17%, n=5) also were significantly more likely to have quit compared to control participants (0%, n=0; χ^2^_1_=5.3, *P*=.02). We noted no difference in cessation rates for males or heavy smokers. Participants experienced significant technology problems during the study. Some participants received duplicate text messages at least once during the trial; others failed to receive some program messages. Neither receiving duplicate messages (χ^2^_1_=0.12, *P*=.73), or missing 5 or more program messages (χ^2^_1_=0.75, *P*=.39) negatively affected quitting rates.

**Conclusions:**

Although the study was not powered to detect statistically significant differences, as the primary aim was to provide estimates of effect size that could be used to better inform a power analysis for a larger trial, findings provide optimism that SMS Turkey may be able to affect quitting rates in environments with high smoking prevalence, such as Ankara, Turkey. The SMS Turkey software program did not work as well as it did 2 years previous. The system will need to be updated to maintain software compatibility with ongoing technology evolution.

**Trial Registration:**

Clinicaltrials.gov NCT00912795 http://clinicaltrials.gov/ct2/show/NCT00912795 (Archived by WebCite at http://www.webcitation.org/6Ch1cIA8l).

## Introduction

Cigarette smoking is a major contributor to morbidity and mortality in Turkey [1,2]. Compared to the United States, where 23% of men and 18% of women are current smokers [3], an estimated 44% of men and 12% of women smoke daily in Turkey [2]. Despite Turkey’s high smoking prevalence rate, data suggest a demand for cessation services—over half of all smokers desire to quit and 45% have made a quit attempt in the past year [2].

The smoking landscape changed dramatically in Turkey when it became the third country in Europe to go 100% smoke free in 2009 [4,5]. Turkey is a signatory of the World Health Organization (WHO)’s Framework Convention on Tobacco Control [6], which mandates the adoption of governmental policies that reduce the supply and demand for tobacco. Turkey has been lauded for its recent success in increasing its efforts to reduce smoking [4,7]. Pharmacotherapies for cessation, such as Zyban, are available at pharmacies without a prescription and a national telephone quit line was implemented in the last few years. However, there is some indication that few smokers avail themselves of cessation services. Unalacak [8] reports that only 3% of current smokers used a smoking cessation intervention (eg, nicotine replacement therapy and cognitive behavioral therapy, CBT) as part of their quit attempt.

To increase cessation rates, smoking cessation programs need to be easily accessible and to reach a large number of people. An estimated 84% of adults in Turkey own a cell phone, 64% of whom use text messaging [9]. Because cell phones are 3.8 times more common than landline telephones [10], text messaging-based programming may represent an underutilized public health opportunity that is both scalable and cost effective [11,12]. Emerging evidence generally supports the efficacy of text messaging-based health behavior change programs [13,14]. This evidence also specifically supports the efficacy of text messaging-based smoking cessation programs in Western countries, at least in the short term [15,16]. Despite these promising data, research is lacking from non-Western cultures and those with higher smoking prevalence rates where the relative morbidity and mortality rates are higher. Unlike in the United States, where tobacco use is considered a “hardening of the target” [17], smoking is normative and very much a social experience in Turkey [18-20]. If text messaging-based programs can be as effective in these high-prevalence settings, the potential public health benefits will be even greater.

Preliminary data from Ankara, Turkey suggests that text messaging-based smoking cessation programs are feasible and acceptable [21,22]. In this paper, we report findings from the small-scale randomized controlled trial (RCT) of short message service (SMS) Turkey, a 6-week text messaging-based smoking cessation program. Given the relative novelty of conducting text messaging-based public health efforts in the Middle East, we also report process measures, including technology issues experienced during the trial and program retention.

## Methods

### Overview

This study was a parallel-group RCT conducted in Ankara, Turkey. Chesapeake IRB and Hacettepe University Ethical Committee reviewed and approved the research protocol. The clinical trial registration number is: NCT00912795.

### Participants

Participants were daily smokers 18 years of age and older living in Ankara, Turkey. Additional eligibility criteria included: owning a mobile phone and having sent or received at least 1 text message in the past year; seriously thinking about quitting in the next 15 days; and not having a chronic or serious illness defined as emphysema, heart disease, or lung disease (because this population would likely require a different type of intervention).

### Study Setting

As the capital of Turkey, Ankara is the second largest city in Turkey after Istanbul. The city is in the heart of the Anatolian peninsula and is part of a main trading route for tobacco [23]. It is estimated that at least 1 smoker resides in 70% of the houses in the southeastern region of Anatolia, which is similar to rates in the country as a whole [24]. In Ankara, 41% of adults are smokers, which ranks the city third in smoking prevalence behind Istanbul (44%) and Izmir (44%) [25]. Ankara’s high smoking prevalence is characteristic of many cities in the Middle East.

### Intervention and Control Group Design

As reported elsewhere [26], the content of the SMS Turkey program was developed following a review of components found in telephone-based counseling approaches to smoking cessation, particularly those using CBT [27-33]. CBT content focuses on altering the individual’s way of thinking (cognitive processes) and acting (behavioral actions). Smokers are encouraged to identify new behaviors that can be substituted for smoking-related activities, make a commitment to quitting, recognize the harmful effects of continued smoking, identify methods to control cues that may trigger the urge to smoke, and reward themselves for not smoking [34]. Self-efficacy theory [35-38] and relapse prevention [28,39-41] are additional components key to an effective smoking cessation program. SMS Turkey integrates these topics into the content and is tailored to where participants are in the quitting process. For example, messages in the “pre-quit” phase encourage the participant to clarify reasons for quitting and to understand his or her smoking patterns and tempting situations/triggers/urges (Table 1). Messages in the “early quit” phase talk about common difficulties and discomforts associated with quitting and emphasize the use of coping strategies. Messages in the “late quit” phase encourage participants to recognize relapse in a different way (eg, situations, confidence, etc) and provide actionable information about how to deal with issues that arise as a non-smoker (eg, stress, moods). Development activities and content were “frozen” and did not change for the life of the trial.

**Table 1:** Example of SMS Turkey content received by the intervention group (actual messages translated into Turkish).

Program arm	Example text message
Pre-quit	When and why do you smoke? Start a smoking diary. Keep track of when you smoke, what you're doing (the activity), how you feel, and your craving (from 1-3).
Quit day	Withdrawal symptoms are unique to everyone. Frustration, impatience, and depression are common but usually only last a week or two.
Early quit	Treat every day like your quit day. Pretend as though it is the first day without cigarettes and be ready for temptation.
Late quit	Call your “special supporter” and make plans for your 2-week anniversary—it's just 3 days away!
Relapse	Becoming a non-smoker is like learning to ride a bike—it's hard at first and then you learn how to do it—and enjoy the ride!
Encouragement	Whatever you decide about smoking, believe in yourself. You CAN quit smoking if you put your mind to it and have a plan for success.

Previous research into the efficacy of telephone quit lines conducted in the United States suggests that most smoking relapse occurs within 2 days of quitting, and at 7 days, the relapse curve begins to flatten out [32]. As such, different content “paths” were created for participants based on whether or not they were smoking 2 days after quit day; and again at 7 days after quit day. If participants reported smoking at either 2 or 7 days after quit day, the research assistant (RA) manually assigned the participant to the “relapse” arm, which provided content that focused on helping them get back on track and recommit to quitting. If participants were smoking at both 2 and 7 days after quit day, they were directed to the “encouragement” arm that focused on norms for quitting and suggested that the person try again when she or he was ready.

Intervention participants began receiving program messages the day after enrollment and continued to receive messages daily through the end of the program. The frequency that participants received messages changed over the course of the program: participants generally received 5 messages per day in the pre-quit phase and then received more messages as the quit day approached. The highest number of messages was sent on the quit day and the day after; and then the number of messages began to taper down. In the last week of the program, participants were sent 1 message per day. Depending on the participant’s content path, the total number of messages received ranged from 91 (for those assigned to the encouragement arm) to 146 (for those who relapsed and then were assigned to the late quit messages).

Intervention messages were created in English, translated into Turkish, and then back-translated to ensure an accurate and appropriate translation. Messages were unidirectional: participants received but did not respond to messages. Research staff did not prompt or remind participants to engage with the intervention.

Previous text messaging-based smoking cessation trials have included a minimal contact control group that received 1 text message per week reminding them they were in the study [16,42]. Control participants in the SMS Turkey RCT were given general quitting information in a 7-page brochure, but they did not receive any text messages. Although the brochure was not designed to exactly mirror the content of the SMS intervention, some information overlapped (eg, setting a quit date, creating a diary to understand their smoking behavior, practicing quitting, and coping strategies for withdrawal). For example, the following text was included in the “It’s Quitting Time” section of the brochure: “First, set a quit date in the next 30 days. Tell everyone when you’re going to stop smoking. Sign a contract and put it on the fridge so that you see it every day. If you smoke 10 or more cigarettes each day, make an appointment with your doctor to talk about medicines that will really help you quit smoking. They may cost money, but think about all of the money you spend on cigarettes!” The brochure encouraged smokers to follow 5 steps: (1) set a quit day and sign a contract, (2) find out about their smoking patterns-why they smoke, (3) practice quitting and change their patterns, (4) involve their family and friends, and (5) learn to be a self-supporter.

### Outcomes

The primary outcome measure was sustained abstinence 3 months after quit day, confirmed with a carbon monoxide (CO) reading of 8 ppm or less [43]. Sustained abstinence was defined as 5 or fewer cigarettes smoked since the quit date, per West et al [44]. Participants were asked: have you smoked at all, even just a puff, since your quit day? Response options were: (1) no, not a puff, (2) 1-5 cigarettes, and (3) more than 5 cigarettes. CO was measured by the RA, who was trained by the project physician (SE) to use the CO device to produce a valid measurement.

Secondary outcome measures included: 7-day and 28-day point prevalence of smoking behavior at 3 months; CO-verified 7-day point prevalence at 4 weeks; and reduction in cigarettes per day for those who are smoking at 3 months. Program acceptability measures included how well intervention participants liked the program and how likely they were to recommend it to others. Participants were also asked if they had suggestions to improve the program and, if so, what the improvement would be.

Covariates included smoking behavior, quitting characteristics, and psychosocial characteristics.

### Smoking Behavior

Participants provided information about their smoking history (eg, age at time of first cigarette) and smoking dependence [45]. Perceptions and norms related to smoking were queried using items developed for research among Turkish smokers [46]. Participants were also asked questions about how different triggers (eg, stress, when someone offers you a cigarette) affected their smoking and about how difficult it was not to smoke in various situations (eg, when with friends) [46].

### Quitting Characteristics

At baseline, participants were asked how important quitting was and how confident they were that they would be able to quit smoking [47]. They also reported quit attempts in past years that lasted for 24 hours or longer, and whether or not they planned to use an evidence-based quitting aid (eg, pharmacotherapy). Reasons for quitting (eg, for family) were queried and a sum was created to reflect the total number of reasons each person had for quitting [46]. Similarly, a sum of 10 different concerns about quitting (eg, I will be more stressed) was created to reflect the total number of concerns participants had about quitting [46]. Finally, we created a summary of good (2 items) and bad (6 items) things about quitting (eg, I will be proud when I quit smoking; I will be less social when I quit smoking).

### Psychosocial Characteristics

Social support is a significant factor in successful cessation efforts [48]. The Multidimensional Scale of Perceived Social Support [49] has 3 subscales: friends, a “special person”, and family (eg, my family really tries to help me). Alcohol dependence is associated with decreased likelihood of cessation [50]. We used the 4-item CAGE measure of alcohol use. CAGE is an acronym for the four questions: (1) have you ever felt the need to *C*ut down, (2) have people *a*nnoyed you by complaining about your drinking, (3) do you ever feel *G*uilty about drinking, and (4) have you ever felt you needed a drink the first thing in the morning (an *E*ye-opener) [51]. We coded endorsement of at least 1 of the 4 drinking-related experiences queried as “problem drinking.”

### Sample Size

We targeted a sample size of 150 participants for feasibility reasons based on the project budget and timeline.

### Randomization and Masking

Participants chose 1 of 2 identical mailing envelopes. Inside, a slip of paper read either “SMS Turkey” (intervention group) or “brochure” (control group). Neither the participant nor the researcher knew which slip of paper was in each envelope.

An imbalance favoring the intervention arm was detected after approximately 100 participants were enrolled. The procedure was then modified so that the RA pulled a slip of paper from a hat that read either “SMS Turkey” or “brochure.” To ensure an equal number of participants in each arm, the number of slips of paper was equal to the number of places that remained in the intervention and control groups.

Participants were told that researchers had developed 2 different programs to help people quit smoking and that the aim of the study was to see if the programs help people quit. The intervention of interest was not specified. Once allocated to a particular arm, neither the RA nor the participant was blind to the participant’s arm assignment.

### Procedures

Participants were recruited and randomized between December 14, 2010, and June 16, 2011, through in-person outreach at local shopping malls and advertisements in local newspapers. Additionally, flyers were posted at Hacettepe University. Smokers indicated their interest by either calling the study office or speaking directly with the RA at the shopping mall. An in-person meeting was then scheduled, during which the RA explained the study, confirmed eligibility criteria, obtained informed written consent, and collected baseline data. The RA also set the participant’s quit day to be 15 days after enrollment. This time frame was chosen to align with the 14-day pre-quit phase for those assigned to the intervention group, although a quit day was set for all participants regardless of their arm assignment. Given that participants needed to be seriously thinking about quitting in the next 15 days to be eligible, this quit date seemed to be acceptable to all participants. The RA encouraged all participants who smoked 10 cigarettes or more per day to consider pharmacotherapy, regardless of their study arm.

Participants had contact with a human being during enrollment, at 2 and 7 days after their quit day (intervention participants only), at data collection follow-ups, and whenever there were technology problems (intervention participants only). Research incentives are not culturally normative in Turkey, so we did not use them in our study.

### Data Collection

The RA collected self-reported survey data and CO readings, which detect cigarette smoking in the previous 24 hours, at the study office at baseline, at 4 weeks after quit day, and at the 3-months follow-up point. We measured program acceptability among intervention participants at 4 weeks. This time point was chosen over the 3-month follow-up point so that participants would have a stronger memory of the program experience.

Participants completed the survey online in a private room at the study office. The survey was pilot tested for validity when delivered online prior to the RCT: 75 adult smokers completed the survey online and 75 completed a paper-and-pencil version of the survey. Responses were similar across mode (unpublished data). If the participant could not come to the office at follow-up, the RA queried smoking status over the telephone by asking the same question included in the survey.

### Statistical Analyses

Analyses were presented in 2 ways. Intent-to-treat (ITT) analyses included all randomized individuals in the analysis (all participants lost to follow-up were assumed to still be smoking). Per-protocol analyses (PPA) included only participants who completed the follow-up measures. It should be noted that PPA is a self-selected sample. Therefore, results are no longer an unbiased sample from a randomized trial. Non-responsive (ie, decline to answer) replies to variables included in the analyses are imputed using best-set regression [52]. All variables have less than 5% of data imputed. We used the “cs” command in Stata to calculate the risk ratio and risk difference [52]. Research suggests that the quitting process may be different for males and females [53] and for heavy (20+ cigarettes per day) versus light smokers [54], so we stratified the sample by each of these 2 characteristics and examined cessation rates by study arm. Finally, to maximize data and therefore increase power, we used a marginal model with generalized estimating equations (GEE) to estimate the population-average odds of CO-verified quitting across the 2 follow-up periods (4 weeks and/or 3 months) as a function of being in the intervention versus control group, while accounting for clustering in the data within person over time. We assumed an exchangeable correlation is assumed and calculated robust standard errors. Baseline characteristics that differed significantly between the intervention and control groups were included in the GEE models. These adjusted estimates are denoted as aOR (adjusted Odds Ratios). All analyses were conducted using Stata 11 [52].

## Results

**Figure 1:**
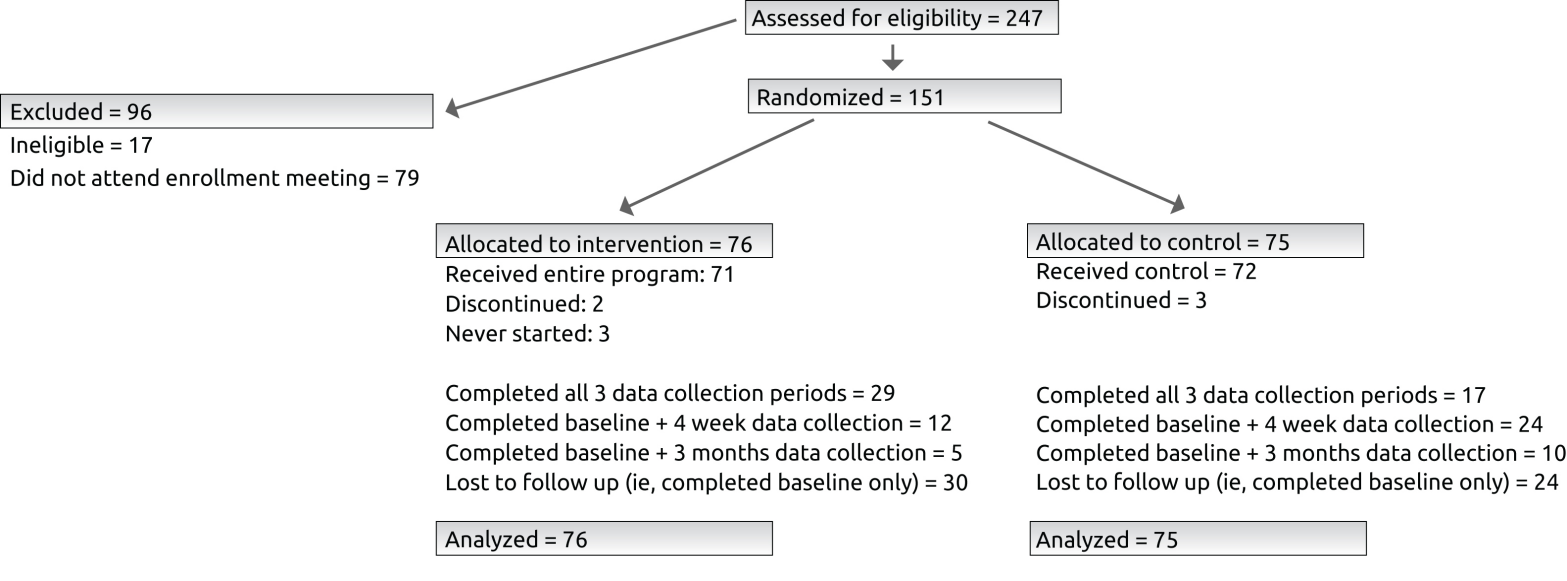
SMS Turkey randomized controlled trial profile.

As shown in Figure 1, of the 247 people who expressed interest in participating, 230 were eligible for the study. Reasons for ineligibility included living outside of Ankara and having a serious health condition. There were 79 people who enrolled in appointments but did not attended. A total of 151 adults (66% of those eligible) attended the enrollment meeting, where they consented to take part in the research study and were randomly assigned to either the intervention or control group.

As shown in Table 2, the experimental groups were generally well balanced on demographic, smoking, and quitting characteristics. Exceptions were the control participants who were significantly more likely to report a low household income, have fewer smoking triggers, have fewer difficulties not smoking when faced with triggers, and identify less strongly with negative things associated with smoking. Conversely, intervention participants reported significantly higher social support from their family and a “special person” in their lives than control participants.

**Table 2:** Sample characteristics by study arm (n=151).

Personal characteristics		Control (n=75)	Intervention (n=76)	Statistical comparison	*P* value
		Mean (SD) or % (n)	Mean (SD) or %( n)		
**Demographic** **characteristics**					
	Age (range 19-62 years)	35.6 (10.3)	36.1 (9.5)	*t*_*149*_=-0.30	0.76
	Female	32.0% (24)	46.1% (35)	χ^2^_1_=3.1	0.08
	Low income (< 2000 Turkish lira per month)	49.3% (37)	30.3% (23)	χ^2^_1_=5.7	0.02
	Married	65.3% (49)	55.3% (42)	χ^2^_1_=1.6	0.21
	Low educational attainment (high school education or lower)	50.7% (38)	36.8% (28)	χ^2^_1_=2.9	0.09
**Smoking** **characteristics**					
	Average number of cigarettes smoked per day (range 7-40)	20.4 (9.2)	18.7 (7.2)	*t*_*149*_=1.2	0.21
	Age at first cigarette (range 6-29 years)	17.1 (3.6)	17.5 (3.7)	*t*_*149*_=-0.71	0.48
	Fagerström score (range 0-10)	4.9 (2.5)	4.8 (2.3)	*t*_*149*_ =0.27	0.79
	Smoking triggers (range 17-65)	38.3 (9.2)	41.9 (7.3)	*t*_*149*_=-2.7	0.01
	Difficulty not smoking when faced with stressors (range 13-45) [46]	32.4 (7.8)	34.8 (5.8)	*t*_*149*_=-2.2	0.03
	Good things about smoking (range 3-15) [46]	5.8 (2.2)	5.9 (2.7)	*t*_*149*_=-0.43	0.66
	Bad things about smoking (range 8-40) [46]	33.1 (8.0)	35.5 (6.2)	*t*_*149*_=-2.1	0.04
	Narghile smoking (ever in the past year)	24.0% (18)	25.0% (19)	χ^2^_1_=0.02	0.89
	Smoker living in the household	48.0% (36)	42.1% (32)	χ^2^_1_=0.5	0.47
**Quitting** **characteristics**					
	Importance of quitting to self (range 4-10) [47]	9.0 (1.4)	8.9 (1.6)	*t*_*149*_=0.37	0.71
	Confidence in one's ability to quit (range 0-10) [47]	6.0 (2.5)	6.0 (2.4)	*t*_*149*_=0.00	0.99
	Number of quit attempts in the past year (range 0-5+)	2.4 (1.5)	2.4 (1.5)	*t*_*149*_=-0.09	0.93
	Number of reasons to quit (range 1-8)	2.7 (1.7)	2.9 (1.6)	*t*_*149*_=-0.81	0.42
	Number of concerns about quitting (range 1-10)	3.5 (2.0)	3.9 (1.9)	*t*_*149*_=-1.3	0.20
	Good things about quitting (range 2-10) [46]	7.6 (1.9)	8.2 (1.7)	*t*_*149*_=-1.8	0.07
	Bad things about quitting (range 6-29) [46]	18.3 (5.1)	18.8 (4.8)	*t*_*149*_=-0.64	0.52
	Planning on using a evidence-based quitting aid	34.7% (26)	31.6% (24)	χ^2^_1_=0.2	0.69
**Psychosocial** **characteristics**					
	Social support from a “special person” (range 4-20)	14.9 (4.0)	16.1 (3.6)	*t*_*149*_=-2.0	0.05
	Social support from family (range 4-20)	15.7 (3.5)	16.8 (3.2)	*t*_*149*_=-2.0	0.05
	Social support from friends (range 4-20)	15.4 (3.1)	16.3 (3.2)	*t*_*149*_=-1.8	0.07
	Problem drinking	29.3% (22)	40.8% (31)	χ^2^_1_=2.2	0.14

### Cessation Results

At 4 weeks, 78% (n=59) of intervention group and 80% (n=60) of control group participants provided cessation data. In addition, 54% (n=41) of intervention group and 55% (n=41) of control group participants provided CO data (χ^2^_1_=0.008, *P*=.93). Data for the 12-week cessation and CO were available for 40% (n=61) of participants: 45% (n=34) intervention group and 36% (n=27) control group (χ^2^_1_=1.2, *P*=.27).

Three-month cessation rates, based upon ITT analyses, were statistically similar for the 2 arms: 11% intervention group versus 5% control group (χ^2^_1_=1.4, *P*=.24; R^2^ = 2.0, 95% CI 0.62-6.3, Table 3). Results were similar when analyzed per protocol: 24% (n=8) in the intervention group versus 15% (n=4) in the control group (χ^2^_1_=0.72, *P*=.40; R^2^=1.6, 95% CI 0.53-4.70). ITT-based population average odds of quitting were similar for those in the intervention group versus control group (aOR=1.7, 95% CI 0.72-4.04).

**Table 3:** Primary and secondary outcomes of the SMS Turkey trial.

		PPA				ITT analysis			
		Control (n=27) % (n)	Intervention (n=34) % (n)	Relative risk (95% CI)	Risk difference (95% CI)	Control (n=75) % (n)	Intervention (n=76) % (n)	Relative risk (95% CI)	Risk difference (95% CI)
**Primary outcome**									
	CO-verified sustained abstinence at 3 months	15 (4)	24 (8)	1.6 (0.53-4.7)	0.09 (-0.11-0.28)	5 (4)	11 (8)	2.0 (0.62-6.3)	0.05 (-0.03-0.14)
**Secondary outcomes**									
	CO-verified 7-day point prevalence abstinence at 4 weeks^a^	12 (7)	15 (9)	1.3 (0.52-3.3)	0.04 (-0.09-0.16)	9 (7)	12 (9)	1.3 (0.50-3.2)	0.03 (-0.07-0.12)
	Self-reported 7-day point prevalence abstinence at 3 months	15 (4)	29 (10)	2.0 (0.70-5.6)	0.15 (-0.06-0.35)	5 (4)	13 (10)	2.5 (0.81-7.5)	0.08 (-0.01-0.17)
	Self-reported 30-day point prevalence abstinence at 3 months	15 (4)	24 (8)	1.6 (0.53-4.7)	0.09 (-0.11-0.28)	5 (4)	11 (8)	2.0 (0.62-6.3)	0.05, (-0.03-0.14)

^a^4-week PPA n=119 (ie, the 59 intervention and 60 control participants who provided cessation data at 4-weeks)

### Investigation of Cessation Results by Important Subpopulations

When the sample was stratified by biological sex (Table 4), ITT-based quitting rates were similar for male intervention group (7%, n=3) and control group participants (8%, n=4; χ^2^_1_=0.009, *P*=.93). Among females, however, intervention group participants (14%, n=5) were significantly more likely to have quit at the 3-month point than control group participants (0%, n=0; χ^2^_1_=3.7, *P*=.05). Population averaged odds suggested that intervention group females were 4.5 times more likely to quit than control group females (95% CI 1.2-16.0), but no differences were noted for males (aOR=0.54, 95% CI 0.12-2.3). Data also suggested that among light smokers, intervention group participants (17%, n=5) were significantly more likely to have quit compared to control group participants (0%, n=0; χ^2^_1_=5.3, *P*=.02). Population averaged odds of quitting were over 4 times higher for light smokers in the intervention group versus control group, but the estimate was not significant (aOR=4.04, 95% CI 0.87-18.6). We did not note a difference in cessation rates for heavy smokers (aOR=0.63, 95% CI 0.16-2.6). Females were significantly more likely than males to be light smokers (58% versus 27%, respectively; *P*<.001), suggesting considerable overlap between females and light smokers.

**Table 4:** Effect of SMS Turkey intervention on the primary outcome by subgroup.

		PPA			ITT analysis		
		Control (n=27) % (n)	Intervention (n=34) % (n)	Statistical comparison	Control (n=75) % (n)	Intervention (n=76) % (n)	Statistical comparison

**Biological sex**							
	Males (n=92)	24 (4)	17 (3)	χ^2^_1_=0.26, *P*=.61	8 (4)	7 (3)	χ^2^_1_=0.009, *P*=.93
	Females (n=59)	0 (0)	31 (5)	χ^2^_1_= 3.9, *P*=.05	0 (0)	14 (5)	χ^2^_1_=3.7, *P*=.05
**Smoking intensity**							
	Light smokers (n=59; < 20 cigarettes per day)	0 (0)	33 (5)	χ^2^_1_=3.8, *P*=.05	0 (0)	17 (5)	χ^2^_1_=5.3, *P*=.02
	Heavy smokers (n=92; 20+ cigarettes per day)	22 (4)	16 (3)	χ^2^_1_=0.25, *P*=.62	9 (4)	7 (3)	χ^2^_1_=0.15, *P*=.69

### Outcomes for Participants Still Smoking at Study End

Among the 47 participants who provided data and were smoking at the 3-month follow-up, the average number of cigarettes smoked daily by intervention group participants (mean 11.9 cigarettes, SD 7.7) was lower, but not significantly so, compared to that reported by control group participants (mean 16.5, SD 9.9; *t*_*43*_=1.8, *P*=.09). On average, participants who were still smoking at follow-up reduced their daily cigarette consumption from baseline to the 3-months point by 5 cigarettes. However, the reduction in cigarettes was not significantly different for intervention group (mean 5.7, SD 7.3) versus control group participants (mean 4.5, SD 8.9; *t*_*43*_=0.51, *P*=.61).

### Intervention Acceptability

The intervention group had 2 people actively drop out: 1 no longer wanted to be in the program and 1 was unreachable because the phone number changed. The control group had 3 people drop out: 2 because they no longer wanted to be in the program and 1 was unreachable because the phone number changed.

Of the 59 intervention group participants who responded at the 4-week follow-up, 69% (n=41) said they somewhat or strongly liked the program and 78% (n=46) were somewhat or very likely to recommend the program to others. When asked what the ideal number of text messages per day would be, the average answer was 5.5 (SD 3.8, range 1-20). The most common suggestion to improve the program was to provide in-person contact, followed by the idea to provide psychological support. Other ideas included talking more about both the benefits of quitting and the dangers of smoking.

### Technology Performance

The software program used to deliver the SMS Turkey program was developed in 2009. Despite functioning well for the pilot feasibility study [22], software challenges were severe enough by the end of the RCT that 2 participants who were randomized to the intervention group could not start the program because the messaging system had failed and could not be resolved. Additionally, 1 person randomized to the intervention group had a phone that was incompatible with the text messaging software program and could not receive messages.

We encountered 2 serious issues with the software program during our study. First, the software program failed to send at least 1 program message to 58% (n=44) of intervention group participants. Most of the affected participants (64%) missed fewer than 5 intervention messages. Intervention participants who missed 5 or more program messages were somewhat less likely than those experiencing fewer interruptions to have a CO-verified smoking status at 3-months: 5% (n=1) vs 12% (n=7; χ^2^_1_=0.75, *P*=.39).

Second, 66% (n=50) of intervention participants were sent a duplicate text message at least once during the trial. Half (50%) of these participants received 22 or more duplicate messages (range 1-342 duplicate messages). Quitting rates were similar for intervention participants who received any number of duplicate text messages versus those who did not receive duplicate messages (11% versus 9%, respectively; χ^2^_1_=0.12, *P*=.73). Furthermore, receiving duplicate messages during one’s quit day–which may be more disruptive in the quitting process–was unrelated to smoking status at 3 months: 12% of those who received duplicate messages within 2 days of their quit day versus 12% of those who received duplicate messages at some other time in the program had quit at follow-up (χ^2^_1_=0.0001; *P*=.99). Six participants were particularly affected and received over 100 duplicate messages. Two of these participants received over 300 messages within a 24-hour period. Unexpectedly, the quit rate among these 6 participants was significantly higher than that for other participants receiving duplicate messages (50% vs 7%, respectively; χ^2^_1_=9.3, *P*=.002).

## Discussion

Despite the public health need to disseminate cost-effective, evidence-based smoking cessation programs, there is a paucity of research regarding the efficacy of these types of smoking cessation programs in Turkey and other countries with cultures that differ from the Western world. If we are to reduce smoking-related morbidity and mortality on a global level, this knowledge is critical in settings with high smoking prevalence rates, such as Turkey [55,56]. Findings from the small-scale RCT of SMS Turkey suggest that the intervention has the potential to affect quitting rates at the 3-month point for women and light smokers who live in Ankara and use text messaging. Results need to be replicated in a well-powered RCT before conclusions can be drawn. Given that literature suggests that males and females have different quitting experiences [53], as do heavy and light smokers [54], understanding which subpopulations may benefit most from these types of cessation services is an important aspect of the larger public health efforts to create an arsenal of evidence-based smoking cessation services that together can meet the varied needs of adult smokers who want to quit.

It is possible that other factors aside from the intervention content affected cessation rates. For example, although participants were told that 2 potentially equal programs were being tested, they may have been able to surmise that the text messaging-based program was the program of interest. If true, then perhaps participants in the text messaging-based program were more motivated and those in the brochure-based program were less motivated to quit. Because both groups received information about quitting, it seems equally possible that the participants believed the explanation that neither program was known to be better and therefore did not have expectations that the brochure should be inferior. Another potential influence on behavior may have been the interaction between the intervention participant and RA at 2 and 7 days after quit day. Even though the RA simply inquired about the participant’s smoking status, this check-in itself could have had some therapeutic effect. Indeed, it may be that text messaging programs that include brief human interaction have enhanced results. This should be studied further.

The SMS Turkey software program did not work as well as it did during the 1-arm feasibility pilot 2 years’ previous. However, it is interesting to note that indicators of program acceptability in this RCT are similar to those found in the previous study [22]. No changes were made to the software program between the time of the feasibility pilot and this RCT. Indeed, the lack of change likely led to the problems. Both our SMS gateway service and our remote server provider updated their software program several times during the course of the seven months we were in field. By June 2011, the software program had stopped functioning altogether and the last 2 people allocated to the intervention group never received program messages. These challenges affected our participants. These problems also affected our recruitment rates because we had to pause recruitment several times to resolve the issues and get the messaging system back on track. Technology is a cost-effective tool that has the promise of widely delivering public health programming. Nonetheless, our experience demonstrates the need to ensure infrastructure to keep this technology up to date. Ongoing technology evolution means that constant updating is necessary to keep software compatible.

Of the eligible participants, 34% did not attend the initial enrollment meeting. Perhaps they did not show up because they were no longer interested in the program or reassessed their readiness to quit smoking. It is possible, however, that they were interested but could not attend because of other commitments. Subsequent trials should consider offering an online enrollment option to investigate whether this option increases the enrollment rate among eligible smokers. Also, the 40% response rate at the 3-month point is suboptimal. This response rate likely reflects the burden of needing to go to the study office to complete study measures. It may also represent the disengagement by intervention group participants who experienced significant technology problems and by control group participants who received minimal study contact. It is possible that this low overall follow-up rate introduced differential bias into the findings, but this seems less likely given that dropout rates were similar between the intervention and control groups. Future trials should consider using follow-up strategies that do not require participants to come to the office (eg, completion of the online survey at home or via text messaging; mail-in saliva *cotinine* tests).

It should be noted that CO tests measure cigarette smoking in the past 24 hours. If participants reported at the 3-month follow-up that they had not had a cigarette since their quit day, but they had actually had a cigarette only a week previous, it would not be detected in the CO test. This limitation would apply equally to control group and intervention group participants, so it is unlikely that it affected the interpretation of the results. Moreover, a review of the literature suggests that biochemical verification is unlikely to change the interpretation of results in minimal contact interventions [57] such as used in this study.

Another important limitation is the study’s small sample size and, therefore, limited power to statistically detect significant differences. As a preliminary RCT, the primary aim was to provide estimates of effect size that could be used to better inform a power analysis for a larger trial. As such, analyses–especially subanalyses–were underpowered. Also, the original randomization technique did not seem to be assigning participants to the study arms equally. Because the 2 arms are balanced on most factors, it appears that allocation concealment was achieved. However, without a visual recording of each enrollment meeting, there is no way to be absolutely certain.

Finally, compared to the national population of smokers in Turkey [2], the study sample was more educated (eg, 32% of smokers in Turkey have a university education, while 56% of trial participants had a university education). Participants in this study also had a profile associated with greater smoking addiction: more had their first cigarette when they were 15 years of age or younger (19% of smokers in Turkey vs 32% of trial participants), more smoked 20 cigarettes a day or more (15% of smokers in Turkey vs 60% of trial participants), and more smoked within 30 minutes of waking (38% of smokers in Turkey vs 57% of trial participants). Future studies should aim to recruit participants with lower educational attainment, and perhaps those with less smoking addiction, to better understand how the program affects smoking cessation in these groups.

### Conclusions

Data from this preliminary RCT provide reason for optimism that SMS Turkey has the potential to affect quitting rates–especially for women and light smokers. These findings provide further support for the hypothesis that, despite their brevity, smoking cessation information can be effectively communicated through a series of 160-character messages. Future research should focus on understanding mechanisms that affect the efficacy of the SMS Turkey program with the aim of eventually including it in the arsenal of evidence-based smoking cessation programs available to Turkish smokers who want to quit.

## Multimedia Appendix 1

CONSORT-eHealth Checklist V1.6 [58].

## Abbreviations

aOR: adjusted Odds Ratio
CBT: cognitive behavorial therapy
CO: carbon monoxide
GEE: generalized estimating equations
ITT: intent-to-treat
PPA: per-protocol analysis
RA: research assistant
RCT: randomized controlled trial
SMS: short message service
WHO: World Health Organization
