# Correction: A Text Messaging-Based Smoking Cessation Program for Adult Smokers: Randomized Controlled Trial

**DOI:** 10.2196/jmir.4655

**Published:** 2015-06-05

**Authors:** Michele Ybarra, A Tülay Bağci Bosi, Josephine Korchmaros, Salih Emri

**Affiliations:** ^1^Center for Innovative Public Health ResearchSan Clemente, CAUnited States; ^2^Hacettepe University, Department of Public HealthAnkaraTurkey; ^3^School of MedicineDepartment of Chest DiseasesHacettepe UniversityAnkaraTurkey

The authors of “A Text Messaging-Based Smoking Cessation Program for Adult Smokers: Randomized Controlled Trial” (*J Med Internet Res* 2012;14(6):e172) have overlooked errors in the Results section during the submission and proofreading process. The percentage of intervention participants who are married should be 55.3% (42) instead of 68.4% (52) in Table 2. The *P* value in Table 4 for Females, ITT analysis, should be *P*=.05; instead of *P*=.53. In the sentence, “Finally, compared to the national population of smokers in Turkey [2], the study sample was more educated (eg, 32% of smokers in Turkey have a university education, while 66% of trial participants had a university education)”, 66% should be changed to 56%. In addition, the corresponding author no longer has a fax number, therefore this has been removed from the original published paper. These errors have been corrected in the online version of the paper on the JMIR website on June 5, 2015, together with publishing this correction notice. A correction notice has been sent to PubMed and the corrected full-text has been resubmitted to Pubmed Central and other full-text repositories.

