# Supplementary material for: A Text Messaging-Based Smoking Cessation Program for Adult Smokers: Randomized Controlled Trial
Source: J Med Internet Res. 2012 Dec 27;14(6):e172. doi: 10.2196/jmir.2231 (PMC3799568; doi:10.2196/jmir.2231)
Supplement: Supplementary file 1 [file jmir_v14i6e172_app1.pdf]

CONSORT-EHEALTH Checklist V1.6 Report

Manuscript Number 2231

Date completed 8/25/2011 14:50:29

by Ybarra

Pilot RCT results of SMS Turkey: a text messaging-based smoking cessation program for adult smokers in Ankara, Turkey

TITLE

1a-i) Identify the mode of delivery in the title

"a text messaging-based"

1a-ii) Non-web-based components or important co-interventions in title

1a-iii) Primary condition or target group in the title

"adult smokers in Ankara, Turkey"

ABSTRACT

1b-i) Key features/functionalities/components of the intervention and comparator in the METHODS section of the ABSTRACT

"randomized either to the 6-week SMS Turkey intervention or brochure control group."

1b-ii) Level of human involvement in the METHODS section of the ABSTRACT

"SMS Turkey messages were sent in an automated fashion except at 2-days and 7-days post Quit day, when the Research Assistant would manually assign participants to content 'paths' based upon whether they were still quit or had relapsed. "

1b-iii) Open vs. closed, web-based (self-assessment) vs. face-to-face assessments in the METHODS section of the ABSTRACT

"self-reported in an online survey"

1b-iv) RESULTS section in abstract must contain use data

"Seventy-six participants were randomly assigned to the intervention, and 75 to the control groups. "

1b-v) CONCLUSIONS/DISCUSSION in abstract for negative trials

"The study was not powered to detect statistically significant differences (as is consistent with pilot designs); findings nonetheless provide optimism that SMS Turkey can affect quit rates in high smoking prevalence environments such as Ankara, Turkey. The SMS Turkey software program did not work as well as it did during the pilot two years previous. Ongoing technology evolution means that constant updating is necessary to keep software compatible. Given that participants who received duplicate messages were less affected in their quit rates compared to participants who missed five or more intervention messages, it seems that the program content may have been helpful in affecting quit rates despite technology problems."

INTRODUCTION

2a-i) Problem and the type of system/solution

"Despite these promising data [for text messaging-based smoking cessation programs in Western cultures], research is lacking from non-Western cultures and those with higher smoking prevalence rates."

"Here, we report findings from the pilot randomized controlled trial (RCT) of SMS Turkey, a 12-week text messaging-based smoking cessation program. Given the relative novelty of conducting text messaging-based public health efforts in the Middle East and the pilot nature of our work, we also report process measures including technology issues experienced and program retention."

2a-ii) Scientific background, rationale: What is known about the (type of) system

"Emerging evidence supports the efficacy of text messaging-based health behavior change programs generally [8, 9] as well as specifically for smoking cessation programs in Western countries – at least in the short term [10]"

METHODS

3a) CONSORT

"Here, we report findings from the pilot randomized controlled trial (RCT) of SMS Turkey, a 12-week text messaging-based smoking cessation program.
"

3b-i) Bug fixes, Downtimes, Content Changes

yes, but in the Results

"The software program used to deliver the SMS Turkey program was developed in 2009. Despite functioning well for the pilot feasibility study [13], two serious issues were encountered with the software program in the current study. First, the software program failed to send at least one program message to 58% (n=44) of intervention participants. Most of the affected participants (64%) missed fewer than five intervention messages. Intervention participants who missed five or more program messages were significantly less likely than those experiencing fewer interruptions to have a CO-verified quit status at 12-weeks: 3% (n=5) vs. 12% (n=12; 2 (1) = 6.2, P = 0.01).
Second, 66% (n=50) of intervention participants were sent a duplicate text message at least once during the trial; half (50%) of these participants received between 22 and 342 duplicate messages. Quit rates were similar for intervention participants who received any number of duplicate text messages versus those who received none (11% versus 9%, respectively, 2 (1) = 0.12, P = 0.73). Furthermore, receiving duplicate messages during one's quit day – which may be more disrupting in the quitting process – was unrelated to quit status at 12-weeks: 12% of those who received duplicate messages within 2-days of their quit day versus 12% of those who received duplicate messages at some other time in the program were quit at follow-up (2 (1) = 0.0; P = 0.99). Six participants were particularly affected and received over 100 duplicate messages, two of whom received over 300 messages within a 24 hour period. Unexpectedly, the quit rate among these six participants was significantly higher than that for other participants receiving duplicate messages (50% vs. 7%, respectively; 2 (1) = 9.3, P = 0.002).
Software challenges were severe enough by the end of the program that two participants who were randomized to the intervention were unable to start the program because the messaging system had failed and could not be resolved.
"

4a-i) Computer / Internet literacy

computer/internet literacy was not required, although text messaging was. this is noted here:

"owning a mobile phone and having sent or received at least one text message in the past year;"

4a-ii) Open vs. closed, web-based vs. face-to-face assessments:

"Self-reported survey data and carbon monoxide readings were collected at the study office at baseline, 4-weeks, and 12-weeks follow-up. Participants completed the survey online in a private room at the study office. If the participant could not come to the office at follow-up, the RA queried smoking status over the telephone by asking the same question included in the survey (see below). "

4a-iii) Information giving during recruitment

"An in-person meeting was then scheduled, during which the RA explained the study, confirmed eligibility criteria, obtained informed written consent, and collected baseline data. "

4b-i) Report if outcomes were (self-)assessed through online questionnaires

"Self-reported survey data "

4b-ii) Report how institutional affiliations are displayed

5-i) Mention names, credential, affiliations of the developers, sponsors, and owners

"Intervention participants began receiving program messages the day after enrollment. Content addressed issues that participants were experiencing as they moved through the stages of quitting. Thus, messages for participants in the pre-quitting phase encouraged the participant to clarify reasons for quitting and to understand one's smoking patterns and tempting situations/triggers/urges (see Table 1). Early Quit messages talked about common difficulties and discomforts associated with quitting, and emphasized the use of coping strategies. Late Quit messages encouraged participants to recognize relapse in a different way (e.g., situations, confidence, etc.) and provided actionable information about how to deal with issues that arise as a non-smoker (e.g., stress, moods).

Previous quit line research in the United States suggests that most smoking relapse occurs within the first 2 days post-quit; at 7 days, the relapse curve begins to flatten out [14]. As such, different content 'paths' were created for participants who were quit versus those who were smoking at 2-days post-quit day; and again at 7-days post-quit day. At either time point, if participants reported smoking, they were pathed to content that focused on helping them get back on track and to recommit to quitting. If participants were smoking at both 2-days and 7-days post-Quit Day, they were pathed to an encouragement arm that focused on norms for quitting and suggested that the person try again when she or he was ready. Messages were uni-directional: participants received but did not respond to messages."

5-ii) Describe the history/development process

the development process is described in other papers, and referenced in teh current paper:
"Preliminary data from Ankara, Turkey suggests that text messaging-based smoking cessation programs are feasible and acceptable "

5-iii) Revisions and updating
"Development activities and content were “frozen” for the life of the trial."
5-iv) Quality assurance methods

5-v) Ensure replicability by publishing the source code, and/or providing screenshots/screen-capture video, and/or providing flowcharts of the algorithms used
we don't provide the content, but we provide examples in Table 1.
5-vi) Digital preservation
see answer above
5-vii) Access
"Participants were recruited through in-person outreach at local shopping malls and advertisements in local newspapers. Additionally, flyers were posted at Hacettepe University. "

"Smokers indicated their interest by either calling the study office or speaking directly with the Research Assistant (RA) at the shopping mall. "

"Research incentives are not culturally normative in Turkey and so were not used in the current study."
5-viii) Mode of delivery, features/functionalities/components of the intervention and comparator, and the theoretical framework
"Intervention participants began receiving program messages the day after enrollment. Content addressed issues that participants were experiencing as they moved through the stages of quitting. Thus, messages for participants in the pre-quitting phase encouraged the participant to clarify reasons for quitting and to understand one’s smoking patterns and tempting situations/triggers/urges (see Table 1). Early Quit messages talked about common difficulties and discomforts associated with quitting, and emphasized the use of coping strategies. Late Quit messages encouraged participants to recognize relapse in a different way (e.g., situations, confidence, etc.) and provided actionable information about how to deal with issues that arise as a non-smoker (e.g., stress, moods). Development activities and content were “frozen” for the life of the trial.

Previous quit line research in the United States suggests that most smoking relapse occurs within the first 2 days post-quit; at 7 days, the relapse curve begins to flatten out [14]. As such, different content ‘paths’ were created for participants who were quit versus those who were smoking at 2-days post-quit day; and again at 7-days post-quit day. At either time point, if participants reported smoking, they were pathed to content that focused on helping them get back on track and to recommit to quitting. If participants were smoking at both 2-days and 7-days post-Quit Day, they were pathed to an encouragement arm that focused on norms for quitting and suggested that the person try again when she or he was ready. Messages were uni-directional: participants received but did not respond to messages."
5-ix) Describe use parameters
messages were sent to the participant daiily; thus the intervention determined the dose
5-x) Clarify the level of human involvement
"At each time point, the RA contacted the participant and asked if they had smoked a cigarette in the past 24 hours, even a puff. At either time point, if participants reported smoking, the RA manually pathed...."
5-xi) Report any prompts/reminders used
"Moreover, participants were not prompted or reminded to engage with the intervention by the research staff. "
5-xii) Describe any co-interventions (incl. training/support)
not applicable
6a-i) Online questionnaires: describe if they were validated for online use and apply CHERRIES items to describe how the questionnaires were designed/deployed
"The online survey was pilot tested prior to the study. 150 adult smokers completed the survey: half online and half via paper-and-pencil. Responses were similar across mode."
6a-ii) Describe whether and how “use” (including intensity of use/dosage) was defined/measured/monitored
usage data, as described above, were not collected.
6a-iii) Describe whether, how, and when qualitative feedback from participants was obtained
intervention participants took part in one-on-one interviews and focus groups. we plan to present these data in a separate paper.
7a-i) Describe whether and how expected attrition was taken into account when calculating the sample size
"A sample size of 150 was targeted for reasons of feasibility based upon the project budget and timeline. "
7b) CONSORT

we did not have any

8a) CONSORT

"Participants chose one of two identical mailing envelopes. Inside, a slip of paper read either ‘SMS Turkey’ (intervention group) or ‘brochure’ (control group). Neither the participant nor the researcher knew which paper slip was in which envelope. An imbalance favoring the intervention arm was detected after about 100 participants were enrolled. The procedure was then modified so that the RA pulled a slip of paper from a hat that read either ‘SMS Turkey’ or ‘brochure’. To ensure an equal number of participants in each arm, the number of slips of paper was equal to the number of ‘slots’ that remained in the intervention and control groups. "

8b) CONSORT

we did not use blocking or stratification.

9) CONSORT

"Participants chose one of two identical mailing envelopes. Inside, a slip of paper read either ‘SMS Turkey’ (intervention group) or ‘brochure’ (control group). Neither the participant nor the researcher knew which paper slip was in which envelope. An imbalance favoring the intervention arm was detected after about 100 participants were enrolled. The procedure was then modified so that the RA pulled a slip of paper from a hat that read either ‘SMS Turkey’ or ‘brochure’. To ensure an equal number of participants in each arm, the number of slips of paper was equal to the number of ‘slots’ that remained in the intervention and control groups. "

10) CONSORT

"Participants chose one of two identical mailing envelopes. Inside, a slip of paper read either ‘SMS Turkey’ (intervention group) or ‘brochure’ (control group). Neither the participant nor the researcher knew which paper slip was in which envelope. An imbalance favoring the intervention arm was detected after about 100 participants were enrolled. The procedure was then modified so that the RA pulled a slip of paper from a hat that read either ‘SMS Turkey’ or ‘brochure’. To ensure an equal number of participants in each arm, the number of slips of paper was equal to the number of ‘slots’ that remained in the intervention and control groups. "

11a-i) Specify who was blinded, and who wasn’t

"Once allocated to a particular arm, neither the RA nor the participant was blind to the participant’s arm assignment."

11a-ii) Discuss e.g., whether participants knew which intervention was the “intervention of interest” and which one was the “comparator”

Participants were told that researchers had developed two different programs to help people quit smoking and that the aim of the pilot study was to see if the programs help people quit; the intervention of interest was not specified."

11b) CONSORT

"Control participants were given a 7-page brochure that provided general quitting information such as setting a quit date, practicing quitting, etc. "

12a) CONSORT

"Logistic regression models were used to estimate the odds of quitting for the intervention versus control groups. "

12a-i) Imputation techniques to deal with attrition / missing values

"Non-responsive (i.e., ‘decline to answer’) responses to variables included in the analyses were imputed using best-set regression [24]. All variables had less than 5% of data imputed. "

12b) CONSORT

"Next, because research suggests that the quitting process may be different for males and females [25], and for heavy (20+ cigarettes per day) versus light smokers [26], the sample was stratified by these two characteristics and cessation rates were again examined by study arm. "

RESULTS

13a) CONSORT

Figure 1

13b) CONSORT

Figure 1

13b-i) Attrition diagram

14a) CONSORT

"Participants were recruited and randomized to either the SMS Turkey intervention or brochure control group between December 14, 2010 and June 16, 2011. "

Follow up assessments were done at 4-weeks and 12-weeks post recruitment

14a-i) Indicate if critical “secular events” fell into the study period

14b) CONSORT

not applicable

15) CONSORT

Table 1

15-i) Report demographics associated with digital divide issues

Compared to the national population of smokers in Turkey, the study sample was more educated (32% vs. 66% had a university education, respectively), and had a profile associated with greater smoking addiction: more had their first cigarette when they were 15 years of age or younger (19% vs. 32%, respectively), more smoked 20 cigarettes a day or more (15% vs. 60%, respectively), and more smoked within 30 minutes of waking (38% vs. 57%)."

16-i) Report multiple “denominators” and provide definitions

"Analyses are presented in two ways: intent-to-treat (ITT) such that all randomized individuals were included in the analysis (all participants lost to follow up were assumed to still be smoking); and per-protocol analysis (PPA) such that only those who completed the follow-up measures were included in the analysis. "

specific n's are reported in the Tables

16-ii) Primary analysis should be intent-to-treat

"Analyses are presented in two ways: intent-to-treat (ITT) such that all randomized individuals were included in the analysis (all participants lost to follow up were assumed to still be smoking); and per-protocol analysis (PPA) such that only those who completed the follow-up measures were included in the analysis. "

17a) CONSORT

see table 2

17a-i) Presentation of process outcomes such as metrics of use and intensity of use

"The software program used to deliver the SMS Turkey program was developed in 2009. Despite functioning well for the pilot feasibility study [13], two serious issues were encountered with the software program in the current study. First, the software program failed to send at least one program message to 58% (n=44) of intervention participants. Most of the affected participants (64%) missed fewer than five intervention messages. Intervention participants who missed five or more program messages were significantly less likely than those experiencing fewer interruptions to have a CO-verified quit status at 12-weeks: 3% (n=5) vs. 12% (n=12; 2 (1) = 6.2, P = 0.01).

Second, 66% (n=50) of intervention participants were sent a duplicate text message at least once during the trial; half (50%) of these participants received between 22 and 342 duplicate messages. Quit rates were similar for intervention participants who received any number of duplicate text messages versus those who received none (11% versus 9%, respectively, 2 (1) = 0.12, P = 0.73). Furthermore, receiving duplicate messages during one’s quit day – which may be more disrupting in the quitting process – was unrelated to quit status at 12-weeks: 12% of those who received duplicate messages within 2-days of their quit day versus 12% of those who received duplicate messages at some other time in the program were quit at follow-up (2 (1) = 0.0; P = 0.99). Six participants were particularly affected and received over 100 duplicate messages, two of whom received over 300 messages within a 24 hour period. Unexpectedly, the quit rate among these six participants was significantly higher than that for other participants receiving duplicate messages (50% vs. 7%, respectively; 2 (1) = 9.3, P = 0.002).

Software challenges were severe enough by the end of the program that two participants who were randomized to the intervention were unable to start the program because the messaging system had failed and could not be resolved.

Beyond technology issues, another process outcome was program retention. Two people actively dropped out of the intervention group: one because they no longer wanted to be in the program and the other because their phone number changed and they were unreachable. Three people dropped out of the control group: two because they no longer wanted to be in the program and one because their phone number changed and they were unreachable. Seventy-eight percent of intervention and 80% of control participants provided cessation data at 4-weeks; 54% and 55%, respectively, also provided CO data (2 (1) =0.008, P = 0.93). Twelve-week cessation and CO data were available for 41% (n=61) of participants: 45% (n=34) intervention and 36% (n=27) control (2 (1) =1.2, P = 0.27).

Finally, appraisal of the program was gathered from intervention participants at 4-weeks post quit. Among the 59 respondents, 69% said they very much or somewhat liked the program; 78% said they would be somewhat or very likely to recommend the program to others.

"

17b) CONSORT

yes, see Table 3

18) CONSORT
"Next, because research suggests that the quitting process may be different for males and females [25], and for heavy (20+ cigarettes per day) versus light smokers [26], the sample was stratified by these two characteristics and cessation rates were again examined by study arm. For a third analysis and to maximize data and therefore increase power, we use a marginal model with generalized estimating equations (GEE) to estimate the population-average odds of CO-verified quitting across the two follow-up periods (4-week and/or 12-week) as a function of being in the intervention versus control group, while accounting for clustering in the data within person over time. An exchangeable correlation was assumed, and robust standard errors calculated. All analyses were conducted using Stata 11 [24]."

18-i) Subgroup analysis of comparing only users
"It should be noted that PPA is a self-selected sample; results are therefore no longer an unbiased sample from a randomized trial."

19) CONSORT
not applicable

19-i) Include privacy breaches, technical problems
"The software program used to deliver the SMS Turkey program was developed in 2009. Despite functioning well for the pilot feasibility study [13], two serious issues were encountered with the software program in the current study. First, the software program failed to send at least one program message to 58% (n=44) of intervention participants. Most of the affected participants (64%) missed fewer than five intervention messages. Intervention participants who missed five or more program messages were significantly less likely than those experiencing fewer interruptions to have a CO-verified quit status at 12-weeks: 3% (n=5) vs. 12% (n=12; 2 (1) = 6.2, P = 0.01).
Second, 66% (n=50) of intervention participants were sent a duplicate text message at least once during the trial; half (50%) of these participants received between 22 and 342 duplicate messages. Quit rates were similar for intervention participants who received any number of duplicate text messages versus those who received none (11% versus 9%, respectively, 2 (1) = 0.12, P = 0.73). Furthermore, receiving duplicate messages during one’s quit day – which may be more disrupting in the quitting process – was unrelated to quit status at 12-weeks: 12% of those who received duplicate messages within 2-days of their quit day versus 12% of those who received duplicate messages at some other time in the program were quit at follow-up (2 (1) = 0.0; P = 0.99). Six participants were particularly affected and received over 100 duplicate messages, two of whom received over 300 messages within a 24 hour period. Unexpectedly, the quit rate among these six participants was significantly higher than that for other participants receiving duplicate messages (50% vs. 7%, respectively; 2 (1) = 9.3, P = 0.002).
Software challenges were severe enough by the end of the program that two participants who were randomized to the intervention were unable to start the program because the messaging system had failed and could not be resolved.
"

19-ii) Include qualitative feedback from participants or observations from staff/researchers

DISCUSSION

20-i) Typical limitations in ehealth trials
"Thirty-four percent of participants who were eligible did not attend the initial enrollment meeting. Perhaps they did not show up because they were no longer interested in the program or reassessed their readiness to quit smoking. It is possible however, that they were interested but unable to attend because of other commitments. Subsequent trials should consider offering an online enrollment option to investigate whether this option increases the enrollment rate among eligible smokers. Also, the 40% response rate at 12-weeks is suboptimal. This likely reflects both the burden of needing to go to the study office to complete study measures; as well as the disengagement by intervention participants who experienced significant technology problems and by the control group who received minimal contact. It is possible that this low overall follow-up rate introduced bias into the findings, but this seems less likely given that dropout rates were similar between the intervention and control groups. Future trials should consider using follow-up strategies that do not require participants to come to the office (e.g., completion of the online survey at home, or via text messaging on one’s cell phone; mail-in saliva continine tests).
Another important limitation is the study’s small sample size and, therefore, limited power to detect statistically significant differences. As a preliminary RCT, the primary aim was to provide estimates of effect size that could be used to better inform a power analysis for a larger trial. As such, analyses – especially subanalyses – were underpowered. Also, the original randomization technique did not seem to be assigning participants to the study arms equally. Although the two arms are balanced on most factors, there are some important characteristics (e.g., income and education) that favor the intervention. Nonetheless, risk rate ratios adjusted the findings for these imbalances.
"

21-i) Generalizability to other populations

21-ii) Discuss if there were elements in the RCT that would be different in a routine application setting

22-i) Restate study questions and summarize the answers suggested by the data, starting with primary outcomes and process outcomes (use)
". Findings from the pilot RCT of SMS Turkey suggest that the intervention group was favored in quitting rates at 12-weeks, although not reliably so. The study was not powered to detect statistically significant differences (as is consistent with pilot designs); findings nonetheless provide optimism that the program can invigorate smoking cessation rates among young adults."

22-ii) Highlight unanswered new questions, suggest future research
"Future research should focus on understanding mechanisms that affect SMS Turkey program efficacy with the aim of eventually including it in the arsenal of evidence-based smoking cessation programs available to Turkish smokers wishing to quit."

Other information

23) CONSORT
Clinical trial registration: NCT00912795

24) CONSORT
the development process is descrbied in an in-press article, which is cited

25) CONSORT
"The project described was supported by Award Number R01TW007918 from the Fogarty International Center. The content is solely the responsibility of the authors and does not necessarily represent the official views of the Fogerty International Center or the National Institutes of Health."

X26-i) Comment on ethics committee approval
"The research protocol was reviewed and approved by Chesapeake IRB and Hacettepe University Ethical Committee. "

x26-ii) Outline informed consent procedures
"An in-person meeting was then scheduled, during which the RA explained the study, confirmed eligibility criteria, obtained informed written consent, and collected baseline data. "

X26-iii) Safety and security procedures

X27-i) State the relation of the study team towards the system being evaluated
"The authors are both the developers and the evaluators of SMS Turkey."
